# Imaging Techniques for Diagnosis of Thoracic Aortic Atherosclerosis

**DOI:** 10.1155/2016/4726094

**Published:** 2016-02-04

**Authors:** Wouter W. Jansen Klomp, George J. Brandon Bravo Bruinsma, Arnoud W. van 't Hof, Jan. G. Grandjean, Arno P. Nierich

**Affiliations:** ^1^Department of Cardiology, Isala Clinics, Dokter van Heesweg 2, 8025 AB Zwolle, Netherlands; ^2^Department of Clinical Epidemiology, Julius Center for Health Sciences and Primary Care, University Medical Center Utrecht, P.O. Box 85500, 3508 GA Utrecht, Netherlands; ^3^Cardiothoracic Surgery, Isala Clinics, Dokter van Heesweg 2, 8025 AB Zwolle, Netherlands; ^4^Department of Cardiothoracic Surgery, Koningsplein 1, 7512 KZ Enschede, Netherlands; ^5^Anaesthesiology and Intensive Care, Isala Clinics, Dokter van Heesweg 2, 8025 AB Zwolle, Netherlands

## Abstract

The most severe complications after cardiac surgery are neurological complications including stroke which is often caused by emboli merging from atherosclerosis in the ascending aorta to the brain. Information about the thoracic aorta is crucial in reducing the embolization risk for both surgical open and closed chest procedures such as transaortic heart valve implantation. Several techniques are available to screen the ascending aorta, for example, transesophageal echocardiography (TEE), epiaortic ultrasound, TEE A-view method, manual palpation, computed tomography, and magnetic resonance imaging. This paper provides a description of the advantages and disadvantages of these imaging techniques.

## 1. Introduction

Neurological complications including stroke are amongst the dreaded complications of cardiothoracic surgery. The incidence of stroke reported in previous studies varied from 1.6% to 17% [1–7]. This wide range may be explained by two factors. First, it has been shown that different definitions of stroke and different strategies to establish the diagnosis greatly impact the incidence; a structured diagnostic protocol for stroke was shown to result in a doubled incidence [7]. Second, the occurrence of stroke is a multifactorial process, and the individual stroke risk depends on multiple pre- and perioperative factors, which include age, female gender, previous cerebrovascular disease, diabetes mellitus, prior heart surgery, prior vascular surgery, history of pulmonary disease, impaired left ventricular function, type of surgery, CPB time, and aortic atherosclerosis [2, 8–12].

## 2. Role of Aortic Atherosclerosis in Neurological Complications

From a pathophysiological perspective, particulate emboli originating from aortic atheroma have been shown to play a pivotal role in the occurrence of postoperative neurological complications [13–18]. Histopathology of emboli captured with an intra-aortic filter showed that 85% of emboli consisted of fibrous atheroma or cap [19]. Also, an autopsy study including 262 patients who died after cardiac surgery showed that cerebral circulatory disturbances were present in 49% of the brains, which primarily consisted of (micro) infarction, followed by cerebral and subarachnoidal haemorrhage; however, the incidence of generalized hypoxemia was low (1.9–2.7% if heart transplant was excluded) [20]. The cause of death was considered to be of primary cerebral aetiology in 12.9% of patients [20].

Stroke is the most evident clinical characteristic after cerebral embolization, but the occurrence of postoperative cognitive dysfunction (PCD), delirium, and dementia may be associated with cerebral emboli. Indeed, presence of aortic atherosclerosis has been associated with an increased risk of postoperative stroke [2, 9–12], postoperative cognitive dysfunction [21], renal dysfunction [22], and mortality [23, 24].

## 3. Imaging Techniques of the (Distal) Ascending Aorta

### 3.1. TEE

Several guidelines have been developed to recommend the position of perioperative use of transesophageal echocardiography in cardiac surgery. Whereas in earlier guidelines TEE was recommended primarily in more complicated procedures (e.g., valve and dissection) [25–27], more recent guidelines recommend its use in all cardiac and thoracic aortic surgeries [28–31]. Detailed descriptions of the technique and views that should be obtained have previously been described [29, 31–33]. Assessment of the thoracic aorta for atherosclerosis or aortic wall pathology constitutes part of this standard examination.

TEE can be performed after induction of anesthesia and before sternotomy, which offers more time from diagnosis of atherosclerosis to the actual change in the surgical management than epiaortic ultrasound. TEE allows adequate visualization of the proximal ascending aorta and thoracic descending aorta [34, 35]. However, visualization of the distal ascending aorta and its branches is hampered by the so-called blind spot caused by the air-filled trachea which interposes the oesophagus and aorta [36, 37]. A meta-analysis of diagnostic accuracy studies showed that the sensitivity of TEE in the diagnosis of severe atherosclerosis of the DAA was a mere 21% [36]. This caveat of TEE was also recognized in the aforementioned guidelines [28, 29, 36].

### 3.2. Epiaortic Ultrasound

In epiaortic ultrasound (EAU) imaging, accurate visualization of the ascending aorta, both proximal and distal, is possible with the direct application of an echo probe onto the aorta. However, visualization of the arch and the origins of the cerebral vessels is limited by anatomic borders such as the pericardium. This method is considered as the gold standard for visualization of the distal ascending aorta [35]. Its use is recommended in patients with an increased stroke risk, such as patients with prior cerebral or vascular disease, and in patients with evidence of atherosclerosis based on other diagnostic tests [38]. Also, EAU can be used as an alternative test in patients with a contraindication for TEE and has not been associated with complications itself, although some concerns have been raised regarding interference with the surgical field. Since EAU requires sternotomy, it can only be applied in a later stage of the operation compared to TEE [38]. Also optimal EAU visualization of the distal ascending aorta, aortic arch, and its branches is performed with the pericardium still closed since opening of the pericardium will reduce visualization of the arch and its side branches.

Screening for aortic atherosclerosis with EAU has been shown to result in changes of the surgical management in 4.1–31% in patients undergoing cardiac surgery [39–43]. Also, several retrospective cohort studies suggested a reduction in stroke [43, 44] and POCD [45], associated with changes in the surgical management after EAU screening. Djaiani et al. included 113 patients in a randomized comparison of screening for atherosclerosis by TEE with manual palpation or the addition of EAU and found that although the surgical management changed more frequently in the EAU group (12% versus 29%, *p* = 0.025), the incidence of cerebral emboli and cognitive dysfunction was similar in both groups. The most frequent change in the surgical plan was an adjustment of the cannulation site (*N* = 14), followed by distal arch cannulation (*N* = 4), fibrillary arrest (*N* = 3), and change to OPCAB (*N* = 2). However, aortic arch imaging was limited by only TEE monitoring of the distal arch and left subclavian artery, probably resulting in underestimation of atherosclerosis.

Multiple guidelines recommended the use of EAU in (high-risk) cardiothoracic surgery [33, 37, 38]. Its use is diminishing however since TEE is often the preferred test as this allows for continuous monitoring and does not interfere with the surgical procedure [37].

### 3.3. Modified TEE or the TEE A-View Method

A-View (Aortic View) technique has been developed to eliminate this so-called blind spot to be used as an additional diagnostic tool prior to cardiac surgery. A modification of TEE has been shown to accurately diagnose aortic atherosclerosis of the DAA, through the placement of a balloon positioned in the trachea, which provides an echocardiographic window to the aorta after inflation with saline [46–48]. The method allows also visualization of the aortic arch and the origins of the cerebral arteries. Therefore, a complete interrogation of the thoracic aorta and branch vessels can be achieved before surgical incision or sternotomy.

After conventional TEE imaging, during which the thoracic aorta is visualized as good as possible, the A-View balloon is introduced into the trachea and left main bronchus and inflated with saline after preoxygenation of the patient. During a period of apnoea, the remaining part of the thoracic aorta, that is, the distal ascending aorta, aortic arch, and its branches can be visualized [46–48]. Compared to EAU, modified TEE had a good overall diagnostic accuracy (area under the receiver operating curve [AUC] of 0.89) for atherosclerosis of the DAA grade 3 or greater, with a positive predictive value (PPV) of 67% and negative predictive value (NPV) of 97% [48]. Also, the diagnosis improved beyond patient characteristics and conventional TEE imaging [49] (atheromatous disease of the aorta was defined by grading the disease using the Katz classification: Grade 1, normal-appearing intima of the aorta, Grade 2, extensive intimal thickening, Grade 3, sessile atheroma protruding <5 mm into the aorta, Grade 4, atheroma protruding >5 mm, and Grade 5, mobile atheroma) [50].

Compared to EAU, modified TEE has the advantage to be performed before the start of the operation. Important decision time for the surgeon is gained in making the right decision in treatment strategy by discussing the patient risk factors and TEE diagnostic information including atherosclerosis before incision. In 12% of the procedures, surgical adaptions were applied, mostly based on change of cannulation site (38%). Also EAU was frequently added to supplement the modified TEE examination giving a more direct guided visualization of the plaque with a more detailed view due to the high frequency probe used in EAU. Implementing the so-called Isala safety check reduced mortality from 2010 till 2013 each year with 15% in the presumed low risk procedures such as CABG, AVR, and combined AVR-CABG [51].

### 3.4. Manual Palpation

Although manual palpation for aortic plaques is routinely performed, it is well known that presence of atherosclerosis is underestimated using this method with sensitivity of 21% [52–54], which also results in fewer changes in the surgical management compared to EAU (see Section 3.2) [42]. The lesions most likely to be missed are noncalcified plaques, which are on the contrary most likely to cause distal embolization. Furthermore, it is conceivable that the manipulation itself causes plaque disturbance. Therefore, we do not consider manual palpation to be of value in the diagnosis of aortic atherosclerosis.

### 3.5. Computed Tomography

Although this paper focuses on the diagnosis of atherosclerosis during surgery, preoperative screening for aortic atherosclerosis can also be achieved using computed tomography (CT) or magnetic resonance imaging (MRI) [55].

The diagnostic accuracy of computed tomography compared to TEE for the presence of aortic atherosclerosis was studied in 47 stroke patients; CT angiography had low sensitivity (52.6%) compared to TEE with positive and negative predictive values of 84.6% and 75.8%, respectively [56]. Another study similarly showed that presence of aortic atherosclerosis was underestimated with CT imaging compared to EAU, with poor reliability between the two methods (kappa: 0.45) [57]. This would imply that CTA is not a good test to exclude aortic atherosclerosis, which could be related to the limited ability to detect (noncalcified) soft plaques. This hypothesis was not addressed in these studies however. Of note, a smaller study (*N* = 32) showed good correlation between aortic arch atheroma thickness diagnosed with CT and TEE imaging (Pearson's *R*: 0.82) [58].

Although the diagnostic accuracy of CT imaging appears to be inferior to EAU and TEE imaging, its results do have prognostic consequences. A “total plaque burden score” calculated from multidetector-row CT angiography (MDCTA) prior to cardiothoracic surgery was associated with increased all-cause mortality; atherosclerosis located in the ascending but not in the descending aorta was an independent predictor [59]. Another study, which included 141 patients planned for minimally invasive mitral valve surgery without sternotomy, showed that in 30 patients multidetector CT (MDCT) screening resulted in a change in the final approach, primarily because of visualization of aortoiliac atherosclerosis. In 29 patients a (partial) sternotomy was performed, while in one patient surgery was cancelled [60]. Also, a retrospective cohort study using a historical comparison group suggested that implementation of preoperative noncontrast CT screening in patients with an increased stroke risk resulted in a reduction of stroke and mortality [61].

The more timely diagnosis of aortic atherosclerosis with CT compared to (modified) TEE and EAU is an important advantage, as this provides more time to plan changes in the surgical management. Disadvantages however are beside a logistic burden, a nephrotoxic risk, and radiation exposure. Moreover, since CT imaging cannot be performed during surgery, the intraoperative guidance of (subtle) changes in the surgical management and continuous monitoring during surgery are impossible. Therefore, although CT imaging may have an important role in specific procedures (e.g., TAVR or minimally invasive MVR) in which the aortic anatomy is also of importance, (modified) TEE or EAU is preferable for the detection of aortic atherosclerosis.

### 3.6. Magnetic Resonance Imaging

Using MR imaging various aspects of aortic atheroma can be characterized, including fibrous cap, lipid core, and thrombus [62]. Several studies compared the diagnosis of aortic atheroma with MRI to TEE [63, 64]. In 99 patients with cryptogenic stroke, the imaging quality (defined as the percentage of the wall circumference assessable with a high level of confidence) of MRI was shown to be superior compared to TEE in the ascending aorta and aortic arch, which was attributed to air artefacts [64]. A good imaging quality of the ascending aorta was observed in 7% and 73% of interrogations with TEE and MR imaging, respectively (*p* < 0.001), although TEE quality was superior for the descending aorta. Accordingly, magnetic resonance imaging showed more complex plaques compared to TEE in the ascending aorta (13 versus 7, *p* = 0.179), aortic arch (37 versus 11, *p* = 0.003), and descending aorta (101 versus 70, *p* < 0.001).

Despite the advantages of MRI, its use in general practice is limited because of several limitations, including current imaging times, availability, costs, and the lack of intraoperative imaging.

## 4. Conclusion

A complete examination of the thoracic aorta is important to guide surgical decision making in treatment algorithms. Information about the thoracic aorta is crucial in reducing the embolization risk for both surgical open and closed chest procedures such as transaortic heart valve implantation.

All imaging modalities do contribute to diagnostic imaging; however, only echo provides real time imaging during the different phases of treatment. If conventional TEE imaging quality is insufficient, additional screening with modified TEE or epiaortic ultrasound is advised. The choice for either test depends on availability and operator experience. Modified TEE has the advantage to be performed before surgical incision, when changes in surgical management or a crossover to a nonsurgical management can still be considered.
